# The potential role of HIV-specific CD38-/HLA-DR+ CD8+ T cells in viral suppressive activity and cytotoxicity in HIV controllers

**DOI:** 10.1186/1471-2334-14-S2-P64

**Published:** 2014-05-23

**Authors:** Stéphane Hua, Camille Lecuroux, Asier Saez-Cirion, Gianfranco Pancino, Isabelle Girault, Martine Sinet, Olivier Lambotte, Alain Venet

**Affiliations:** 1 INSERM U1012, Le Kremlin Bicêtre, France

## Introduction

In HIV-1 infection, some rare patients called HIV controllers (HICs) are capable to spontaneously control viral replication in vivo. Interestingly, HICs exhibit higher frequency of a particular activated phenotype CD38-HLA-DR+ HIV-specific CD8+ T cells. The aim of this study was to characterize this profile and evaluate its role in HICs.

## Materials and methods

To investigate the functionality of the CD38-HLA-DR+ profile, we compared it with the classically activated phenotype CD38+HLA-DR+ by evaluating several qualitative parameters: (1) activation measured by CD69, CD25, CD71, CD40 and Ki67 expression, (2) memory parameters measured by proliferation capacity, CD127 and Bcl-2 expression, cytokine production measured by IL-2 production and (3) cytotoxic activity. We also determined the mechanism responsible for this particular profile.

## Results

CD38-HLA-DR+ cells exhibited a more resting profile than CD38+HLA-DR+ cells marked by a lower expression of several activation markers. Although they presented similar ex vivo profile especially concerning survival, IL-2 production, CD38-HLA-DR+ cells displayed significantly higher HIV-specific cytotoxic capacity after in vitro culture compared to CD38+HLA-DR+ cells (13% [7%-23%] vs. 7% [3%-11%], p=0.02). Furthermore only the frequency of CD38-HLA-DR+ HIV-specific CD8+ T cells correlated with the capacity of CD8+ T cells to inhibit viral replication ex vivo (r=0.32, p<0.0001). Moreover, the CD38-HLA-DR+ profile was preferentially displayed after activation by low doses of antigen. These results are in line with the enhanced expression of this profile in patients which exhibit high functional sensitivity (r=0.41, p=0.01).

## Conclusions

Collectively, these data highlight the cytotoxic role of CD38-HLA-DR+ expressing HIV-specific CD8+ T cells in HICs and we provide insights into the mechanism of its induction. Induction of this type of protective cell subset could be an important goal in vaccine strategies.

